# An Enhanced Smoothed *L*_0_-Norm Direction of Arrival Estimation Method Using Covariance Matrix

**DOI:** 10.3390/s21134403

**Published:** 2021-06-27

**Authors:** Ji Woong Paik, Joon-Ho Lee, Wooyoung Hong

**Affiliations:** 1 Radar System Team 2, Hanwha Systems, Yongin-City 17121, Korea; jw.paik90@hanwha.com; 2 Department of Information and Communication Engineering, Sejong University, Seoul 05006, Korea; joonhlee@sejong.ac.kr; 3 Department of Defense Systems Engineering, Sejong University, Seoul 05006, Korea

**Keywords:** compressive sensing, direction of arrival, smoothed L0-norm method

## Abstract

An enhanced smoothed $l_{0}$-norm algorithm for the passive phased array system, which uses the covariance matrix of the received signal, is proposed in this paper. The SL0 (smoothed $l_{0}$-norm) algorithm is a fast compressive-sensing-based DOA (direction-of-arrival) estimation algorithm that uses a single snapshot from the received signal. In the conventional SL0 algorithm, there are limitations in the resolution and the DOA estimation performance, since a single sample is used. If multiple snapshots are used, the conventional SL0 algorithm can improve performance in terms of the DOA estimation. In this paper, a covariance-fitting-based SL0 algorithm is proposed to further reduce the number of optimization variables when using multiple snapshots of the received signal. A cost function and a new null-space projection term of the sparse recovery for the proposed scheme are presented. In order to verify the performance of the proposed algorithm, we present the simulation results and the experimental results based on the measured data.

## 1. Introduction

The direction-of-arrival (DOA) estimation method is a basic required technique to estimate the locations of the targets. A representative method to estimate the DOAs of targets uses the phase difference between antennas based on the uniform linear array (ULA). Typical examples of the DOA estimation method are the conventional beamforming algorithm, and adaptive beamforming algorithm, which are beamforming-based methods [1,2,3,4]. The multiple signal classification (MUSIC) algorithm estimates the DOAs of the targets using the orthogonality of the noise part eigenvectors and the steering vectors [5].

The conventional DOA estimation algorithms [1,2,3,4,5,6,7] are based on an overdetermined system. Therefore, the sensor failure of an array becomes a factor that greatly degrades the DOA estimation performance of the algorithms.

In contrast, the compressive-sensing-based DOA estimation methods extend the received signal to an underdetermined system using the spatial sparsity of the incident signals of the targets and estimate the DOAs of the targets through the sparse recovery. Thus, the compressive-sensing-based DOA estimation methods are more robust with the sensor failure than the conventional DOA estimation methods.

In order to estimate the DOAs of the incident signals based on the CS method, a data-fitting algorithm was proposed [8]. At first, a single snapshot measurement vector of a received signal is used for the sparse recovery. In order to enhance the DOA estimation performance of the data-fitting algorithm, the single measurement formulation of the conventional algorithm was expanded to the multiple snapshots measurement formulation. In [9], which is a study on extending the data-fitting DOA algorithm to the bistatic MIMO sonar system is presented, and the high-resolution direction-of-departure (DOD)/DOA algorithm is proposed.

In [10,11,12], a compressive-sensing-based covariance-fitting DOA estimation algorithm was proposed. The amount of computations was proportional to the number of samples when the basis pursuit denoising (BPDN)-based cost function was used. The covariance-fitting method was presented in [10,11,12], and the DOA was estimated using the sparse recovery of the signal covariance matrix. Since the number of entries to be recovered is independent of the number of samples, it is possible to solve the problem of computational complexity due to an increase in the number of samples.

The biggest problem with these algorithms [8,9,10,11,12] is that they are computationally intensive, and they are highly dependent on the noise variance of a given environment. In [8,9,10,11,12], a regularization parameter, which is the weight of the cost function, is highly dependent on the noise variance. If the noise variance that is used for the corresponding parameter does not match the noise variance of the environment, the DOA estimation performance of the corresponding methods is greatly degraded.

The smoothed $l_{0}$-norm (SL0) [13,14] is a method of approximating the discontinuous function $L_{0}$-norm with an arbitrary continuous function. The solution of the $L_{0}$-norm function can be obtained by the global maxima of the corresponding function. This method requires less computations than solving the $L_{1}$-norm minimization problem, and it does not require an accurate noise variance.

In [15], a joint smoothed $l_{0}$-norm-based DOA estimation method in multiple input–multiple output (MIMO) radar system was presented. The proposed scheme in [15] reduces the dimension of the received signal matrix by using the feature of MIMO radar system and the singular value decomposition of the received signal matrix. In [16], a reweighted smoothed $l_{0}$ norm-based DOA estimation method for the monostatic MIMO radar system was proposed. To reduce the computation time, [16] used vectorized diagonal terms of the signal covariance matrix by using the feature of the monostatic MIMO radar system and only considering uncorrelated targets. A weight vector to enhance the accuracy of the scheme is presented in [16] by using the noise subspace which can only be obtained when the number of targets is given in advance. In [17], a robust SL0 approach for MIMO radars was presented to provide accurate angle-range-Doppler estimates. The ill-conditioned problem was studied when applying SL0 for MIMO radar angle-range-Doppler estimates. In [18], an adaptive beamforming based on compressive sensing with the SL0 method was presented. The proposed scheme in [18] can greatly reduce the elements of the array without degenerating the performance of the beams. In [19], a new sparse signal representation model was proposed. The proposed scheme shows robust performance on DOA estimation by using the lower left diagonals of the covariance matrix. In [20], a method which enhances the range-Doppler imaging performance for noise radar was presented by generalized SL0 method. In [21], a regularized weight SL0 minimization method for underdetermined blind source separation was proposed. the proposed scheme in [21] shows the superior performance in signal and image recovery. In [22], a new smoothing modified Newton algorithm based on $l_{p}$ norm regularization (SMN-lp) was presented to recover the sparse signal.

The smoothed $L_{0}$-norm (SL0) method only uses a single snapshot. The gradient descend method is used for the process of mapping a signal vector using a continuous function to find the global maxima, which is in the feasible set.

In order to perform the sparse recovery using a single snapshot, the conventional method is vulnerable to the low SNR and adjacent multiple targets. DOA estimation with multiple snapshots of received signals from correlated targets or a rich multipath environment is practically important.

To enhance the performance of the conventional scheme and robustness for the correlated signal, a covariance-fitting-based SL0 algorithm is presented in this paper. By using the covariance-fitting method, instead of using multiple snapshots, the number of optimization variables for the sparse recovery could be reduced.

The proposed algorithm is considered for use in a passive sonar system. Considering the correlated signal is essential to properly estimate the DOAs of the multiple targets of the measurement data collected underwater.

In this paper, we derived a process of extending the conventional method to enable the covariance fitting and the cost function of the sparse recovery based on covariance fitting is presented. The null-space projection term that adjusts the optimization result so that it is always included in the feasible set is proposed to be suitable for the signal covariance extended to the potential DOA set. In order to verify the performance of the proposed algorithm, we present the simulation results, which are based on MATLAB and the experimental results, which are based on the measured data.

## 2. Signal Model

In order to use the compressive-sensing method, the solution that needs to be obtained must be sparse. The solution of the DOA estimation in the passive sonar system is the incident angle of the received signal. The received signal is the incident on the sensor array only in a specific direction. In other words, the received signal has a spatially sparse feature.

The received signal at $t_{i}$ can be expressed as

(1) $$y\left(t_{i}\right)=\mathrm{Ax}\left(t_{i}\right)+n\left(t_{i}\right).$$

When *p* denotes the number of targets, the signal vector $x\left(t_{i}\right)$ can be written as (2) in the passive sonar system.

(2) $$x\left(t_{i}\right)=\left[\begin{array}{c} x_{1}\left(t_{i}\right)\\ \vdots \\ x_{p}\left(t_{i}\right) \end{array}\right].$$

In order to use the compressive sensing method, the signal vector $x\left(t_{i}\right)$ should be changed as

(3) $$x\left(t_{i}\right)=\left[\begin{array}{c} x_{1}\left(t_{i}\right)\\ \vdots \\ x_{N_{\theta }}\left(t_{i}\right) \end{array}\right],$$

where $N_{\theta }$ is the number of search angles for θ. A denotes an array manifold, and the array manifold in the compressive-sensing method can written as

(4) $$A=\left[\begin{array}{cccc} a\left(\theta_{1}\right) & a\left(\theta_{2}\right) & \cdots & a\left(\theta_{N_{\theta }}\right) \end{array}\right].$$

*M* is the number of sensors. The array vector $a\left(\theta_{n}\right)$ can be written as

(5) $$a\left(\theta_{n}\right)={\left[\begin{array}{cccc} a_{1}\left(\theta_{n}\right) & a_{2}\left(\theta_{n}\right) & \cdots & a_{M}\left(\theta_{n}\right) \end{array}\right]}^{T}.$$

The noise vector $n\left(t_{i}\right)$ is a complex Gaussian random vector whose real parts and imaginary parts of the noise elements are Gaussian-distributed with $N\left(0,\sigma^{2}/2\right)$:

(6) $$n\left(t_{i}\right)={\left[\begin{array}{cccc} n_{1}\left(t_{i}\right) & n_{2}\left(t_{i}\right) & \cdots & n_{M}\left(t_{i}\right) \end{array}\right]}^{T}.$$

## 3. Conventional Smoothed $l_{0}$ Norm Based DOA Estimation

The conventional smoothed $l_{0}$ norm method estimates the DOAs of the target by only using a single snapshot. Using (3), the *i*-th element of $x\left(t_{i}\right)$ mapped to the Gausian function can be expressed as

(7) $$f_{\sigma }\left(x_{k}\left(t_{i}\right)\right)≜\exp\left(\frac{-{\left(x_{k}\left(t_{i}\right)\right)}^{2}}{2\sigma^{2}}\right),\;\;\;k=1,\cdots ,N_{\theta },$$

where sigma is the variance of the Gaussian function. When (7) is defined, (8) is established, and it can be approximated and expressed as (9)

(8) $$\lim_{\sigma \to 0}f_{\sigma }\left(x_{k}\left(t_{i}\right)\right)=\left\{\begin{array}{c} 1,\;\;\;\mathrm{if}\;\;\;\;x=0\\ 0,\;\;\;\mathrm{if}\;\;\;\;x\neq 0 \end{array}\right.$$

(9) $$f_{\sigma }\left(x_{k}\left(t_{i}\right)\right)=\left\{\begin{array}{c} 1,\;\;\;\mathrm{if}\;\;\;\;\left|x\right|\ll \sigma \\ 0,\;\;\;\mathrm{if}\;\;\;\;\left|x\right|\gg \sigma \end{array}\right..$$

Using (3) a continuous multivariate function $F_{\sigma }\left(x\left(t_{i}\right)\right)$ can be defined as

(10) $$F_{\sigma }\left(x\left(t_{i}\right)\right)=\sum_{k=1}^{N_{\theta }}f_{\sigma }\left(x_{k}\left(t_{i}\right)\right).$$

Using Equation (10), we can see that the function $F_{\sigma }\left(x\left(t_{i}\right)\right)$ outputs the number of elements whose value is 0 in the signal vector. Therefore, when the variance of the Gaussian function, σ, is a very small value, the following equation holds:

(11) $${∥x\left(t_{i}\right)∥}_{0}\approx N_{\theta }-F_{\sigma }\left(x\left(t_{i}\right)\right).$$

Using (11), the conventional cost function of the $l_{0}$-norm minimization method can be expressed as

(12) $$\min{∥x\left(t_{i}\right)∥}_{0}\;\;\;\mathrm{subject}\;\mathrm{to}\;\;\;Ax\left(t_{i}\right)=y\left(t_{i}\right)\Rightarrow \max F\left(x\left(t_{i}\right)\right)\mathrm{subject}\;\mathrm{to}\;\;\;Ax\left(t_{i}\right)=y\left(t_{i}\right).$$

The $\min{∥x\left(t_{i}\right)∥}_{0}$ problem of the compressed sensing-based DOA estimation method, which is a discontinuous function, can be expressed by the problem of finding the global maxima of the continuous function through mapping to a Gaussian function. The solution of the cost function expressed by the continuous function is required to find the global maxima by using the gradient descent method.

## 4. Covariance-Fitting Smoothed $l_{0}$ Norm Based DOA Estimation

Let *L* denote the number of snapshots. For i=1,⋯,L in (1), which is the received signal matrix Y, can be written as

(13) Y=AX+N.

The array covariance matrix obtained from the time average can be defined as

(14) $$\hat{R}=\frac{1}{L}\sum_{i=1}^{L}y\left(t_{i}\right)y{\left(t_{i}\right)}^{H}\approx A{\hat{R}}_{x}A^{H}+E.$$

Every element of $R_{X}$ should be optimized using the sparse recovery. The Gaussian function was used in this paper in order to map the elements of the signal covariance matrix. Using (14), the Gaussian function can be written as

(15) $$f_{\sigma }\left({\left(R_{X}^{}\right)}_{k,l}\right)≜\exp\left(\frac{-{\left({\left(R_{X}^{}\right)}_{k,l}\right)}^{2}}{2\sigma^{2}}\right),\;\;\;k,l=1,\cdots ,N_{\theta },$$

where ${\left(R_{X}\right)}_{k,l}$ denotes the entry that lies in row number *k* and column number *l*.

Equation (15) can be approximated to:

(16) $$f_{\sigma }\left({\left(R_{X}^{}\right)}_{k,l}\right)\approx \left\{\begin{array}{cc} 1, & \mathrm{when}\;\left|{\left(R_{X}^{}\right)}_{k,l}\right|\ll \sigma \\ 0, & \mathrm{when}\;\left|{\left(R_{X}^{}\right)}_{k,l}\right|\gg \sigma \;. \end{array}\right.$$

Using (16), $F_{\sigma }\left(R_{X_{}}^{}\right)$ can be defined as

(17) $$F_{\sigma }\left(R_{X}\right)=\sum_{k=1}^{N_{\theta }}\sum_{l=1}^{N_{\theta }}f_{\sigma }\left({\left(R_{X}\right)}_{k,l}\right).$$

By using (16), it is clear that (17) represents the number of zero elements of $R_{X_{}}^{}$ when σ is close to 0.

The cost function of the sparse recovery can be defined as

(18) $$\begin{array}{c} \min{∥R_{X_{}}^{}∥}_{0}\;\\ \mathrm{subject}\;\mathrm{to}\;\;\;\hat{R}={\mathrm{AR}}_{X}^{}A^{H}. \end{array}$$

${∥\cdot ∥}_{0}$ denotes the $l_{0}$ norm which calculates the number of non-zero elements. In order to make $R_{X_{}}$ sparse, $\min{∥R_{X_{}}∥}_{0}$ should be minimized. The $l_{0}$ norm of $R_{X}$ and the expression in (17) has following relation:

(19) $${∥R_{X}^{}∥}_{0}\approx N_{\theta }^{2}-F_{\sigma }\left(R_{X}^{}\right).$$

When σ is close to 0, using (19), the cost function of the sparse recovery can be approximated as

(20) $$\begin{array}{c} \max F_{\sigma }\left(R_{X}\right)\\ \mathrm{subject}\;\mathrm{to}\;\;\;\hat{R}={\mathrm{AR}}_{X}^{}A^{H}. \end{array}$$

Setting the σ value is important in order to obtain $R_{X}$ which is sparse and preserves information about the signal sources. When σ is small, $F_{\sigma }$ has many local maxima and is highly non-smooth, so it is difficult to maximize it. Conversely, if σ is close to being infinite, $F_{\sigma }$ becomes easy to maximize because it is smoother than the previous case and has a lesser local maximum. In order to easily and efficiently maximize $F_{\sigma }$, σ should be set to gradually decrease from *∞* to 0.

Furthermore, making the output of the gradient descent method in the feasible set, which is an important part in order to recover the sparse $R_{X}$. In order to make the output $R_{X}$ of the method in a feasible set, the following process is essential:

(21) $${\hat{R}}_{X}^{}={\hat{R}}_{X}^{}-A_{}^{†}\left({A\hat{R}}_{X}^{}A^{H}-\hat{R}\right){\left(A^{H}\right)}_{}^{†}.$$

**Figure 1:**
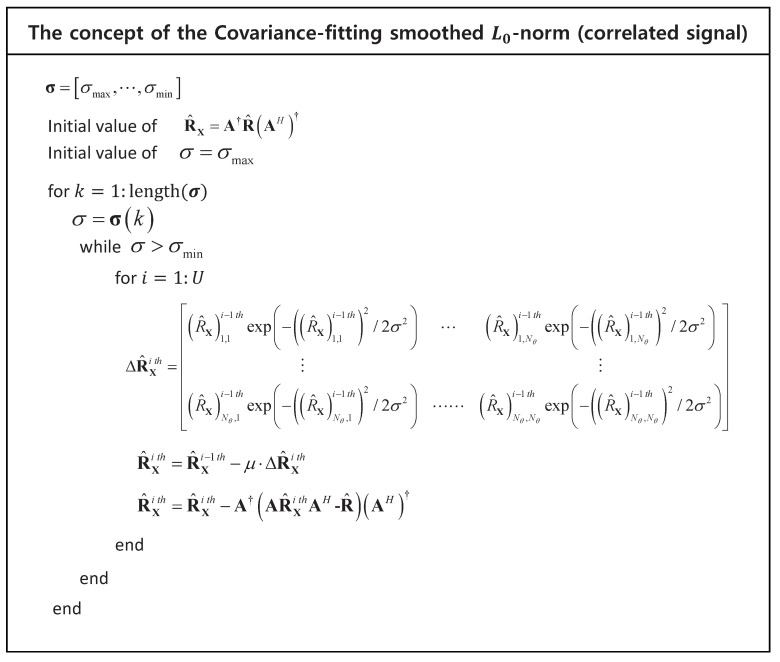
The concept of the covariance-fitting SL0 (correlated signal).

Figure 1 illustrates the concept of the proposed scheme. Using the ${\hat{R}}_{X}={\hat{R}}_{X}-\mu \cdot \Delta {\hat{R}}_{X}$, (21) can be expressed as

(22) $$\begin{array}{c} {\hat{R}}_{X}={\hat{R}}_{X}-A^{†}\left({A\hat{R}}_{X}A^{H}-\hat{R}\right){\left(A^{H}\right)}^{†}\\ \;\;\;\;\;\;\;\;\;\;=\left({\hat{R}}_{X}-\mu \cdot \Delta {\hat{R}}_{X}\right)-A^{†}\left(A\left({\hat{R}}_{X}-\mu \cdot \Delta {\hat{R}}_{X}\right)A^{H}-\hat{R}\right){\left(A^{H}\right)}^{†}\\ \;\;\;\;\;\;\;\;\;\;={\hat{R}}_{X}-\mu \cdot \Delta {\hat{R}}_{X}-A^{†}\left({A\hat{R}}_{X}A^{H}-\mu \cdot A\Delta {\hat{R}}_{X}A^{H}-\hat{R}\right){\left(A^{H}\right)}^{†}\\ \;\;\;\;\;\;\;\;\;\;={\hat{R}}_{X}-\mu \cdot \Delta {\hat{R}}_{X}+\mu \cdot A^{†}A\Delta {\hat{R}}_{X}A^{H}{\left(A^{H}\right)}^{†}\\ \;\;\;\;\;\;\;\;\;\;={\hat{R}}_{X}-\mu \cdot \left(\Delta {\hat{R}}_{X}-A^{†}A\Delta {\hat{R}}_{X}A^{H}{\left(A^{H}\right)}^{†}\right). \end{array}$$

$A^{†}A$ is a projection matrix onto the row space of A. (22) can be written as

(23) $$\begin{array}{c} {\hat{R}}_{X}={\hat{R}}_{X}-\mu \cdot \left(\Delta {\hat{R}}_{X}-A^{†}A\Delta {\hat{R}}_{X}{\left(A^{†}A\right)}^{H}\right)\\ \;\;\;\;\;\;\;\;={\hat{R}}_{X}-\mu \cdot \left(\Delta {\hat{R}}_{X}-A^{†}A\Delta {\hat{R}}_{X}A^{†}A\right). \end{array}$$

Since the result is the same, no matter how many times the projection is executed, Equation (23) can be simplified as

(24) $$\begin{array}{c} {\hat{R}}_{X}={\hat{R}}_{X}-\mu \cdot \left(\Delta {\hat{R}}_{X}-A^{†}A\Delta {\hat{R}}_{X}\right)\\ \;\;\;\;\;\;\;\;={\hat{R}}_{X}-\mu \cdot \left(I-A^{†}A\right)\Delta {\hat{R}}_{X}. \end{array}$$

$\left(I-A^{†}A\right)$ in (24) is a null-space projector. The gradient $\Delta {\hat{R}}_{X}$ is controlled to fit in the feasible set by the null-space projector.

## 5. Numerical Results

In order to verify the DOA estimation performance of the covariance-fitting SL0, the spectra of the proposed scheme are compared with the conventional beamforming algorithm. The structure of the sensor array is set to a uniform linear array. When the value of the wavelength is set, the uniform linear array structure is settled so the inter-element spacing of the receiver becomes half the wavelength.

**Figure 2:**
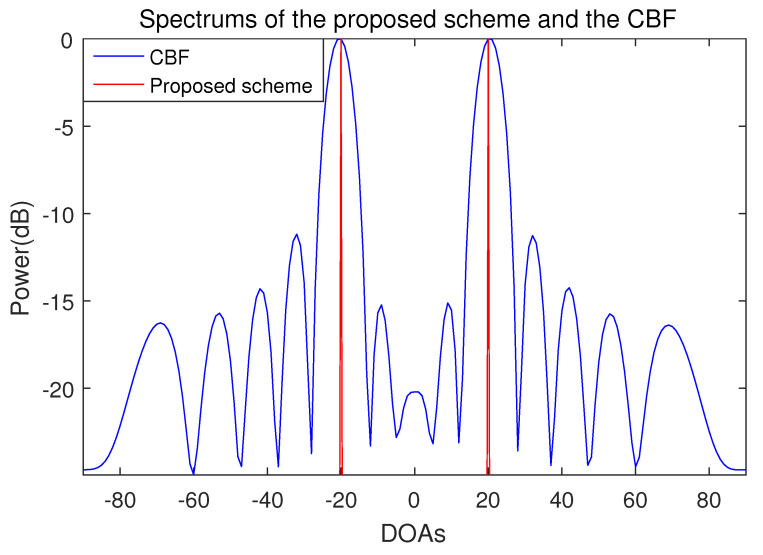
Spectrum of the proposed scheme and the conventional beamforming algorithm when the number of sensors is 15, the number of samples is 1000 and the incident angles are $-20^{{}^{\circ}}$ and $20^{{}^{\circ}}$ (SNR = 10 dB).

**Figure 3:**
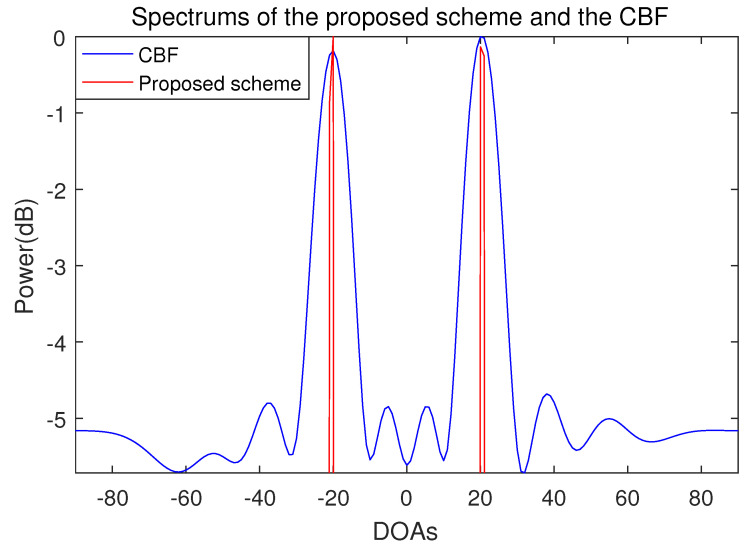
Spectrum of the proposed scheme and the conventional beamforming algorithm when the number of sensors is 10, the number of samples is 500, and the incident angles are $-20^{{}^{\circ}}$ and $20^{{}^{\circ}}$ (SNR = −10 dB).

**Figure 4:**
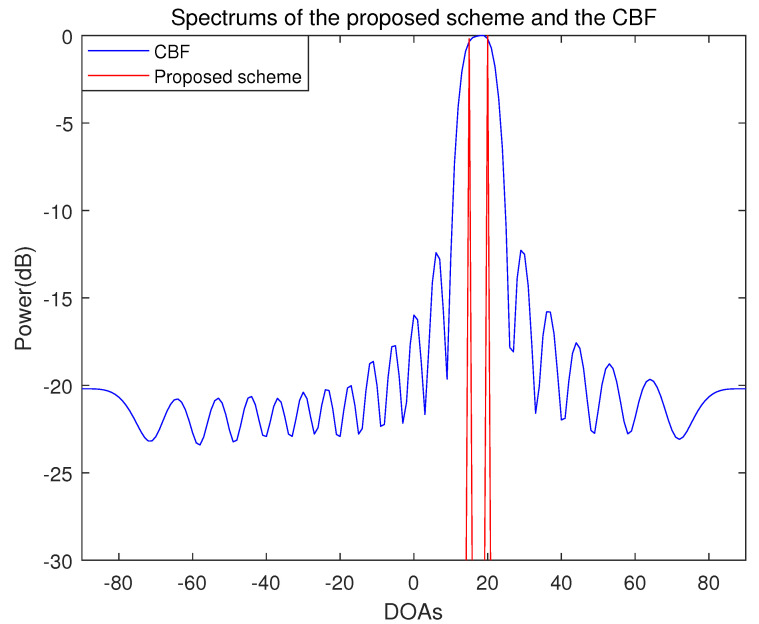
Spectrum of the proposed scheme and the conventional beamforming algorithm when the number of sensors is 20, the number of samples is 500 and the incident angles are $15^{{}^{\circ}}$ and $20^{{}^{\circ}}$ (SNR = 10 dB).

The number of incident signal sources is two and the DOAs of the incident signals are set in the two cases. In case 1, the location of the two incident signal sources are far apart from each other and the DOAs are $-20^{{}^{\circ}}$ and $20^{{}^{\circ}}$. In case 2, the two incident signal sources are adjacent and the DOAs of the two incident signal sources are $15^{{}^{\circ}}$ and $20^{{}^{\circ}}$. The results of the proposed scheme and the conventional beamforming algorithm are shown in Figure 2, Figure 3 and Figure 4.

The spectra of the proposed scheme and the conventional beamforming algorithm when the DOAs of two incident signals are $-20^{{}^{\circ}}$ and $20^{{}^{\circ}}$ are shown in Figure 2 and Figure 3. Both the proposed scheme and the conventional beamforming algorithm accurately estimate the DOAs of the incident signals for all the SNRs. In the conventional beamforming algorithm, the average power of the sidelobes increases as the SNR decreases. When the SNR is −10 dB, the power difference between the mainlobe and the sidelobe is around 5 dB. In contrast to the conventional beamforming algorithm, which shows a typical beam pattern, the spectrum of the only proposed scheme has sharp peaks in the part that corresponds to the DOAs. This is because the compression sensing-based DOA estimation method is not a beamforming method, so the mainlobe and the sidelobe do not exist in the spectrum.

The resolution performance of the proposed scheme and the conventional beamforming algorithm is shown in Figure 4. In this case, the DOAs of two incident signals are $15^{{}^{\circ}}$ and $20^{{}^{\circ}}$. In the conventional beamforming algorithm, it can be seen that the two incident signals cannot be resolved. In contrast to the conventional beamforming algorithm, it can be seen that the proposed scheme accurately resolves the two adjacent incident signals at $5^{{}^{\circ}}$ interval.

**Figure 5:**
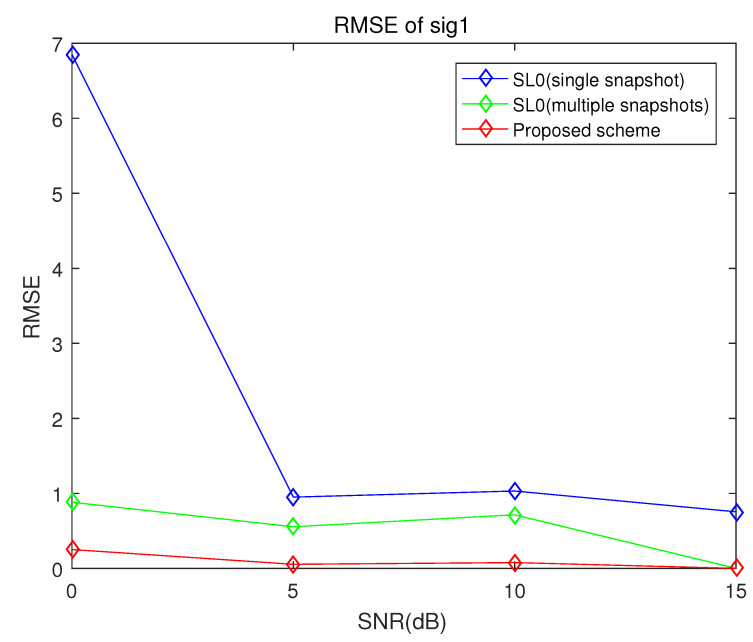
RMSE of the proposed scheme, the conventional smoothed $l_{0}$ norm method and multiple snapshots smoothed $l_{0}$ norm method when the number of sensors is 10, the number of samples is 100 and the incident angles are $5^{{}^{\circ}}$ and $20^{{}^{\circ}}$ (sig1).

**Figure 6:**
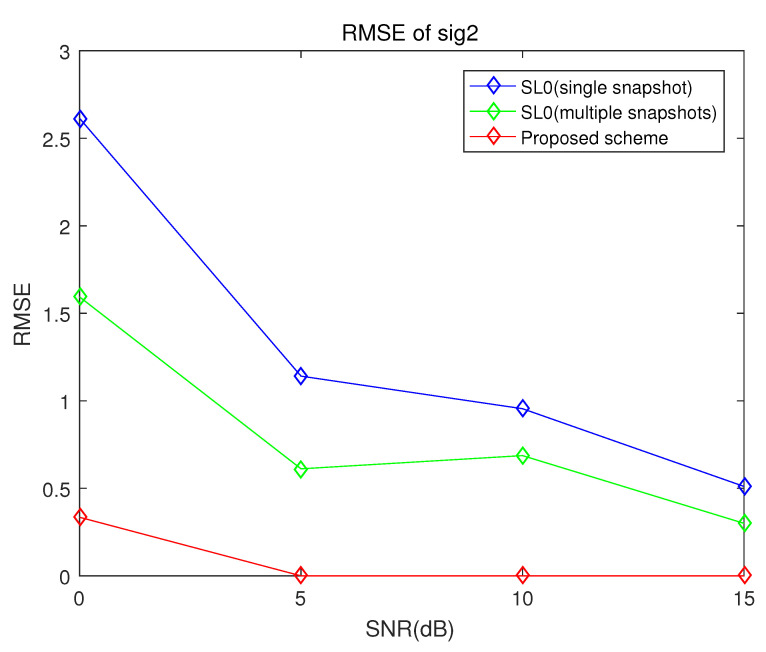
RMSE of the proposed scheme, the conventional smoothed $l_{0}$ norm method and multiple snapshots smoothed $l_{0}$ norm method when the number of sensors is 10, the number of samples is 100 and the incident angles are $5^{{}^{\circ}}$ and $20^{{}^{\circ}}$ (sig2).

Figure 5 and Figure 6 show the root mean square (RMS) errors of the proposed scheme, the conventional smoothed $l_{0}$ norm method and the multiple snapshots-based smoothed $l_{0}$ norm method versus signal-to-noise ratio (SNR) with 10 sensors and 1000 snapshots. The true DOA of the first target is $5^{{}^{\circ}}$ and that of the second target is $20^{{}^{\circ}}$. These simulation results in Figure 5 and Figure 6 verify that the DOA estimation performance of the proposed scheme is superior to that of the conventional smoothed $l_{0}$ norm method and the multiple snapshots-based smoothed $l_{0}$ norm method.

As can be seen in Figure 5 and Figure 6, the proposed scheme and the multiple snapshots-based smoothed $l_{0}$ norm method are robust to noise level. On the other hand, the RMS errors of the conventional smoothed $l_{0}$ norm method increase rapidly when SNR is 0 dB.

**Figure 7:**
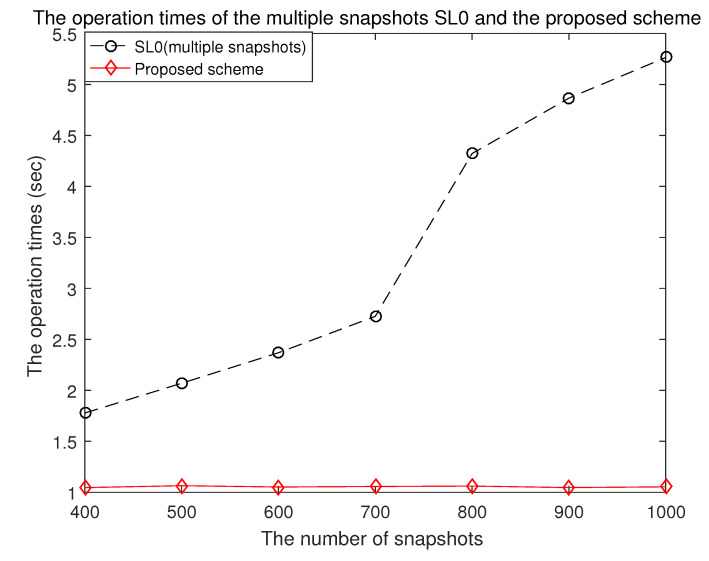
The operation times of the multiple snapshots SL0 and the proposed scheme.

In the case of the proposed scheme, the number of optimization variables can be maintained regardless of the number of snapshots by optimizing the signal covariance matrix extended to the potential DOA set. In Figure 7, the operation times of the proposed scheme and the multiple snapshots-based smoothed $l_{0}$ norm method with respect to the number of snapshots are depicted. In the case of the multiple snapshots-based smoothed $l_{0}$ norm method, as the number of snapshots increases, the number of optimization variables increases. Therefore, the computational complexity significantly increases in proportion to the number of snapshots.

**Figure 8:**
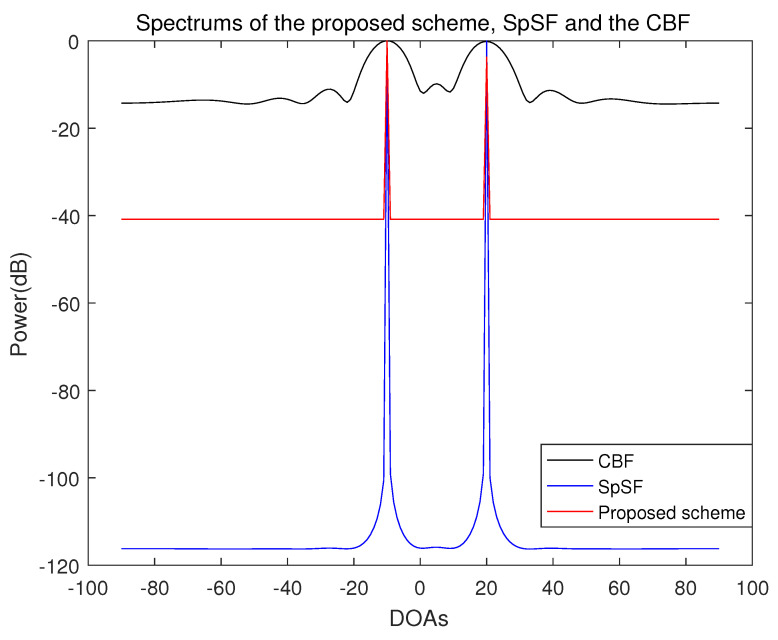
Spectrum of the proposed scheme, SpSF and the conventional beamforming algorithm when the number of sensors is 10, the number of samples is 1000 and the incident angles are $-10^{{}^{\circ}}$ and $20^{{}^{\circ}}$ (SNR = 5 dB).

In Figure 8, the spectra of the proposed scheme, the SpSF(sparse spectrum fitting) algorithm, which is a compressive-sensing-based covariance-fitting DOA estimation using convex optimization, and the conventional beamforming DOA estimation algorithm is presented. Comparing the spectra of the proposed scheme with the spectra of the SpSF algorithm, except for the difference in dynamic range which indicates the power difference between the peaks and the noise floor, there is no significant difference in the DOA estimation performance. However, in terms of computational complexity, there is a distinct difference between the proposed scheme and the SpSF algorithm.

**Figure 9:**
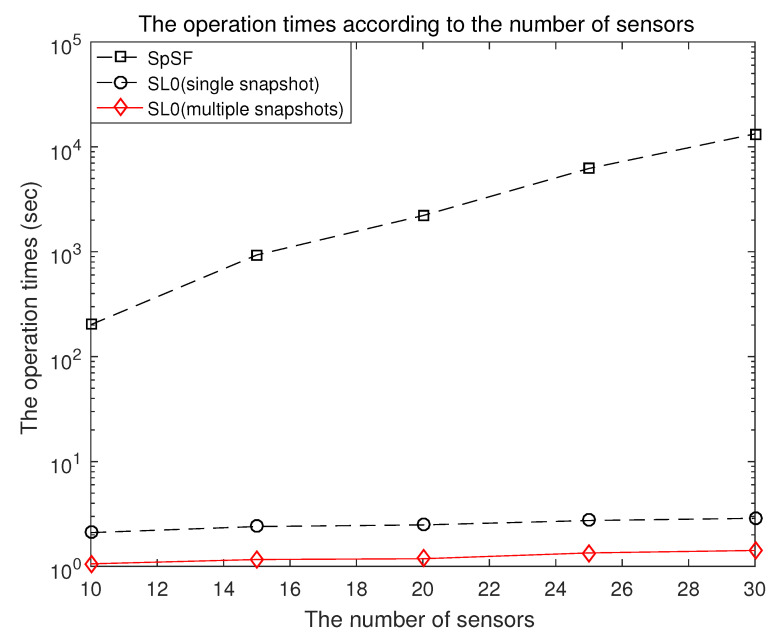
The operation times of the proposed scheme, multiple snapshots SL0 and SpSF.

The operation times of the proposed scheme, the multiple snapshots-based smoothed $l_{0}$ norm method and the SpSF algorithm with respect to the number of sensors are shown in Figure 9. The computational complexity is measured by using the operation time function of MATLAB and the specifications of the computer include an Intel(R) Core(TM) i7-6700 CPU @ 3.40 GHz (Santa Clara, CA, USA). The operation time of the SpSF is 229.25 s. It can be verified in Figure 9 that the computational complexity of the proposed scheme is significantly lower than that of the SpSF. The proposed scheme is more efficient than SpSF by considering both aspects of the performance of DOA estimation and computational complexity.

**Figure 10:**
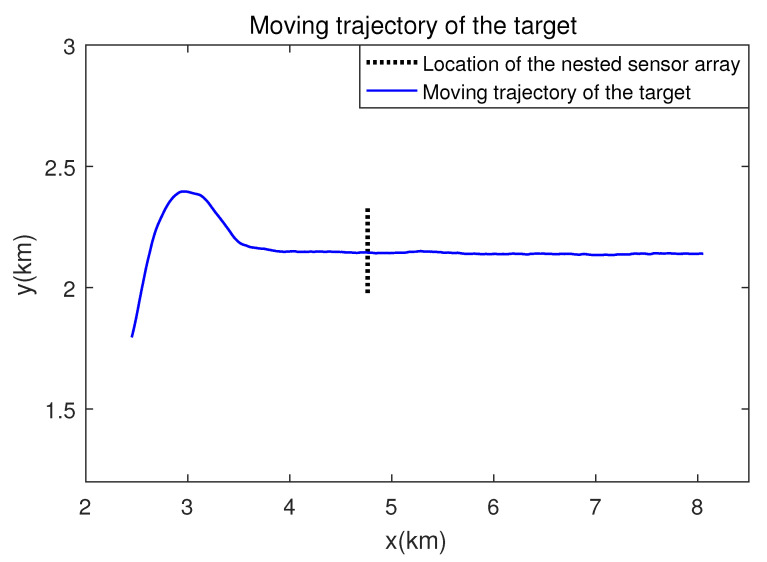
The moving trajectory of the target and the location of the horizontal nested sensor array of the measured data.

For the reliability of the proposed scheme, the experiments were conducted based on the measured data. The measurement data used in this paper are the received data from the underwater horizontal nested sensor array. The moving signal source passes over the nested sensor array, which is shown in Figure 10 and it emits 21 tonal frequencies.

**Figure 11:**
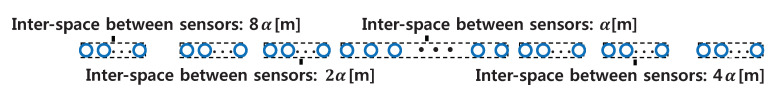
Structure of the horizontal nested sensor array.

The results for one of them is shown in this paper. The received data from the sensor array was collected for 66 min. During the collection, objects other than the moving target to be detected appeared and disappeared. Information, such as the nested sensor array specifications and the design frequencies, is confidential, so every frequency-related piece of information is normalized by a pilot signal frequency, and the structure of the sensor array is shown in Figure 11.

The number of sensors of the underwater horizontal nested array is 120 and the nested sensor array can make a uniform linear sensor array by using 48 sensors for each design frequency. The parameters of the experiments based on measured data can be seen in Table 1.

The DOA estimation experiments, which are based on the measured data, are conducted using the beamforming-based DOA estimation methods (the conventional beamforming algorithm and minimum variance distortionless response (MVDR)), and the compressive-sensing-based covariance-fitting DOA estimation methods, which include the SpSF and the proposed scheme.

The DOA estimation performances of the proposed scheme, which is the SpSF algorithm, the conventional beamforming algorithm and MVDR based on the measured data, are shown in Figure 12, Figure 13, Figure 14, Figure 15, Figure 16, Figure 17, Figure 18, Figure 19, Figure 20, Figure 21, Figure 22 and Figure 23.

Figure 12, Figure 13, Figure 14, Figure 15, Figure 16, Figure 17, Figure 18, Figure 19, Figure 20, Figure 21, Figure 22 and Figure 23 illustrate the bearing time records (BTRs) of the four DOA estimation methods when the signal frequencies are 1 Hz, 0.142 Hz and 0.37 Hz.

The BTR is a figure that sequentially stacks the DOA estimation results of the measured data divided by 10 s. Through the corresponding BTR, the moving paths of the targets, which were detected by the nested sensor array for 66 min, can be estimated. In the SpSF, a noise variance of a received signal should be provided in advance. Therefore, a value of the noise variance for each received datum used for one instance of DOA estimation is obtained through preprocessing.

**Figure 12:**
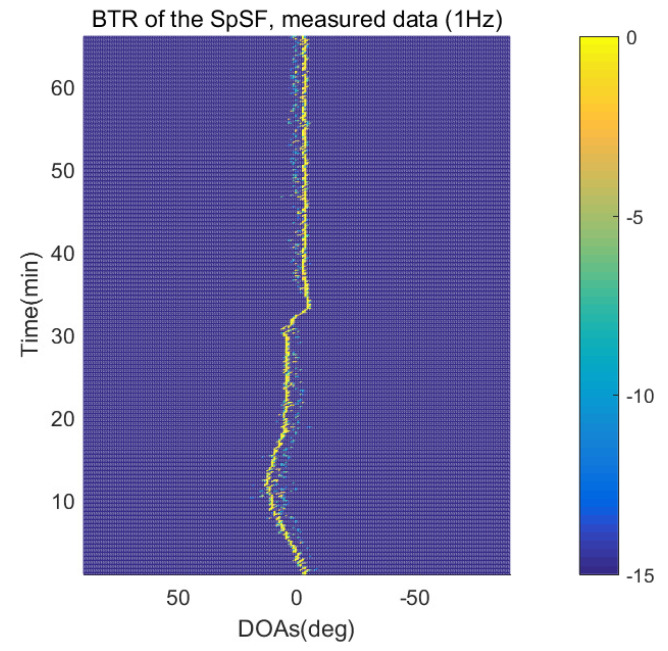
Bearing time records of the SpSF: 66 min, pilot signal frequency = 1 Hz.

**Figure 13:**
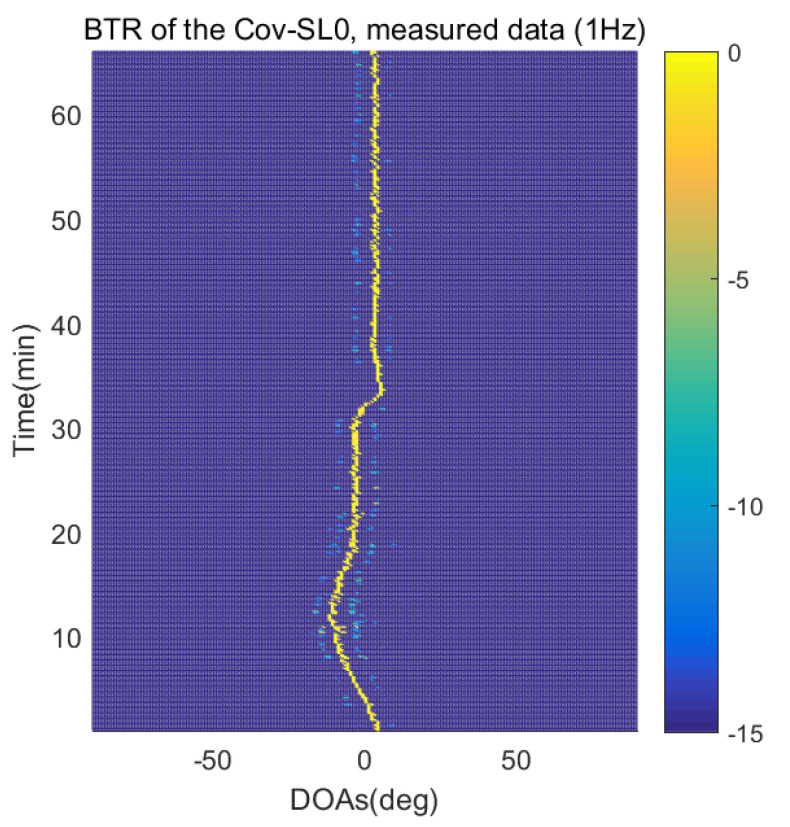
Bearing time records of the proposed scheme: 66 min, pilot signal frequency = 1 Hz.

**Figure 14:**
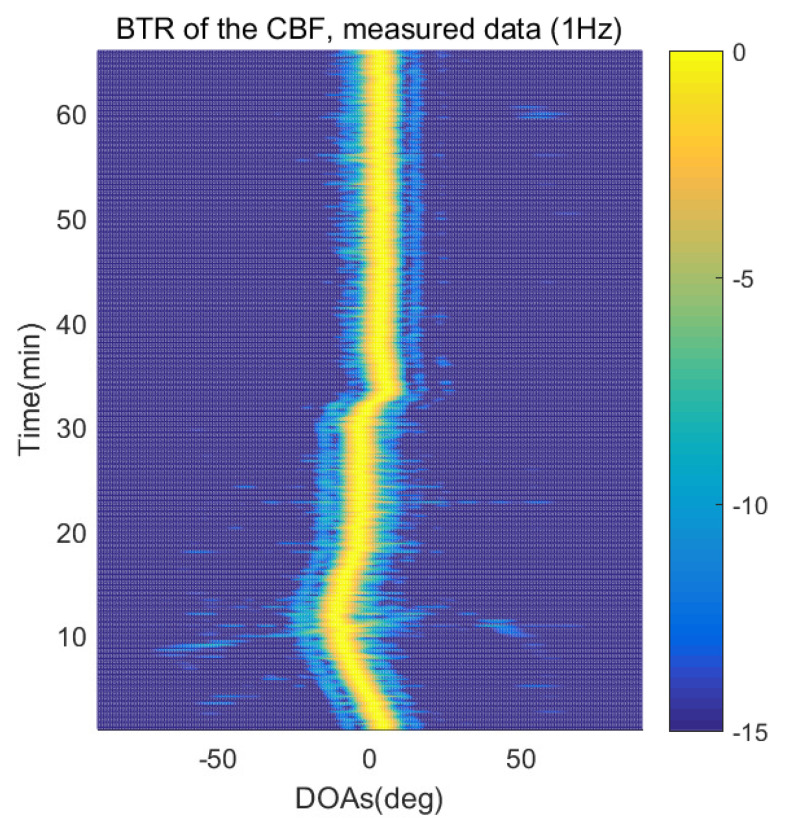
Bearing time records of the CBF: 66 min, pilot signal frequency = 1 Hz.

**Figure 15:**
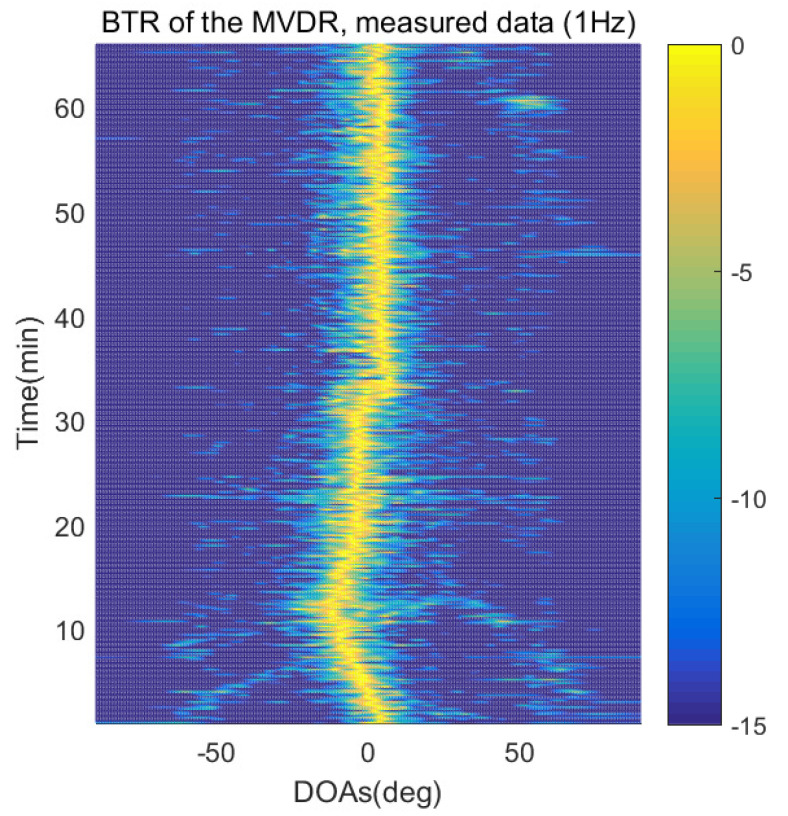
Bearing time records of the MVDR: 66 min, pilot signal frequency = 1 Hz.

Figure 12, Figure 13, Figure 14 and Figure 15 show the case where the pilot signal with an abnormally large signal level is emitted from the moving signal source in order to accurately estimate the moving trajectory of the target using the nested sensor array, so the trajectories of the other ships do not appear in the BTR, but only that of the target. The compressive-sensing-based DOA estimation methods have superior performance than the beamforming-based methods. Therefore, in Figure 12 and Figure 13, the compressive-sensing-based methods show the trajectory of the moving target with higher resolution on the BTR than the beamforming-based methods in Figure 14 and Figure 15. The proposed scheme and the SpSF have practically the same performance regarding the DOA estimation.

**Figure 16:**
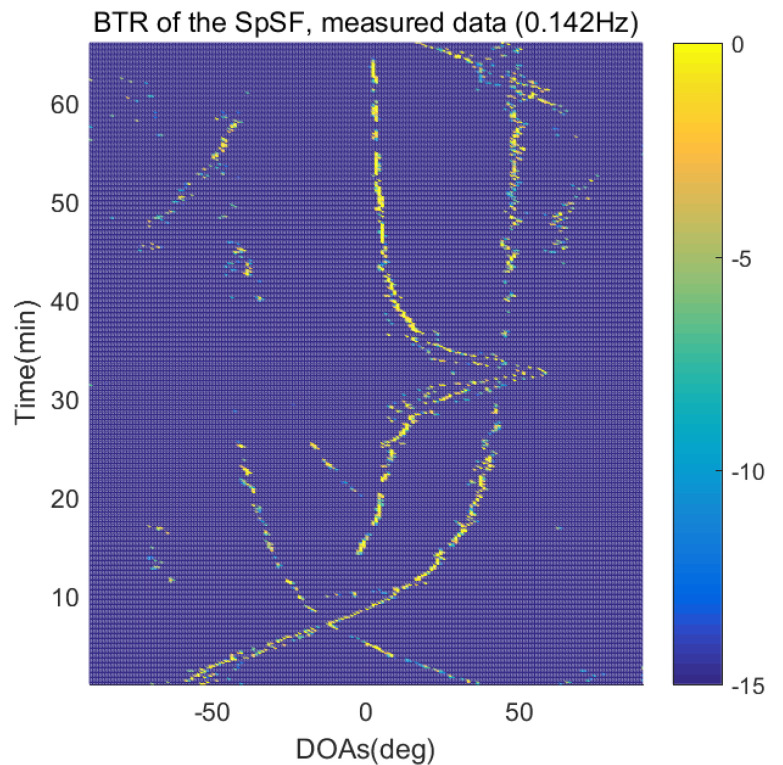
Bearing time records of the SpSF: 66 min, signal frequency = 0.142 Hz.

**Figure 17:**
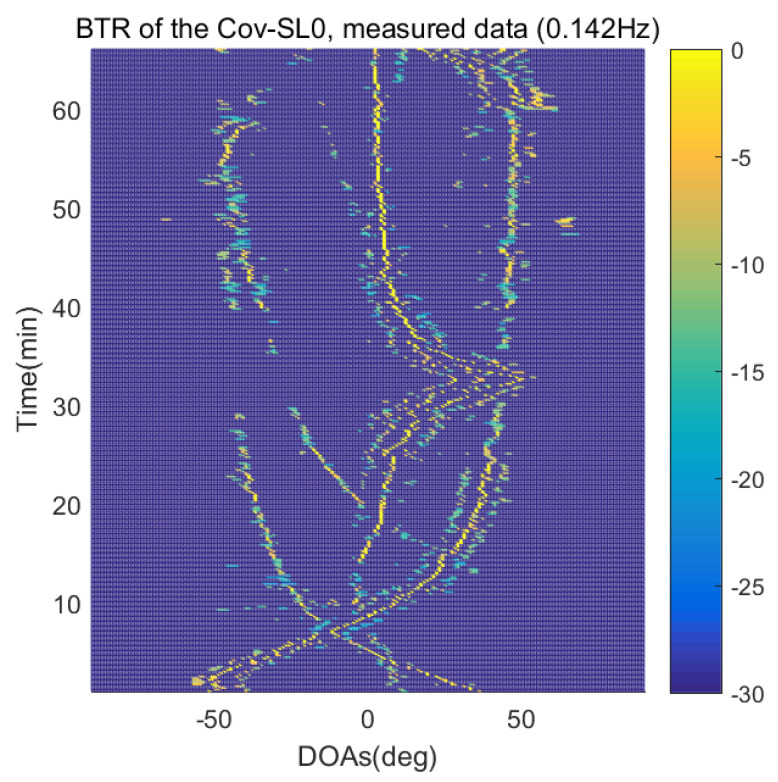
Bearing time records of the proposed scheme: 66 min, signal frequency = 0.142 Hz.

**Figure 18:**
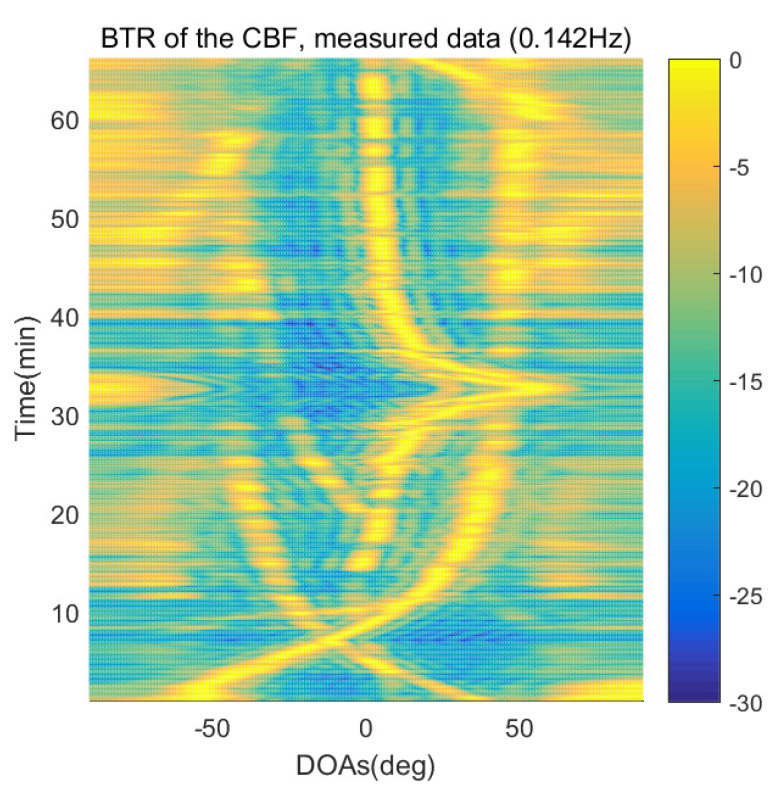
Bearing time records of the CBF: 66 min, signal frequency = 0.142 Hz.

**Figure 19:**
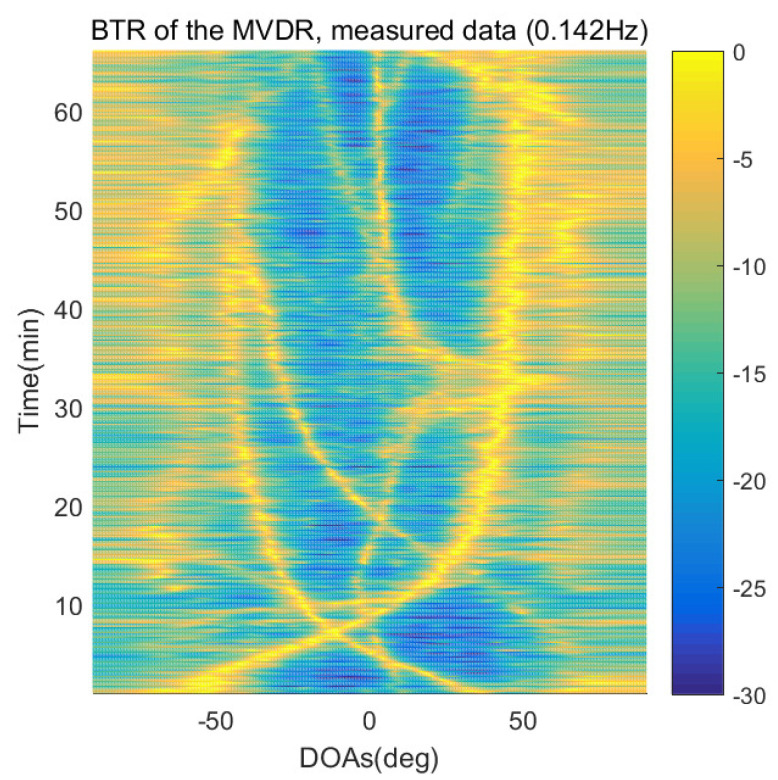
Bearing time records of the MVDR: 66 min, signal frequency = 0.142 Hz.

In Figure 16, Figure 17, Figure 18 and Figure 19, the performance of the compressive-sensing-based covariance-fitting methods and the beamforming-based methods are shown when the signal frequency, which is 0.142 Hz, has a signal level that is 10 dB lower than the pilot signal frequency.

**Figure 20:**
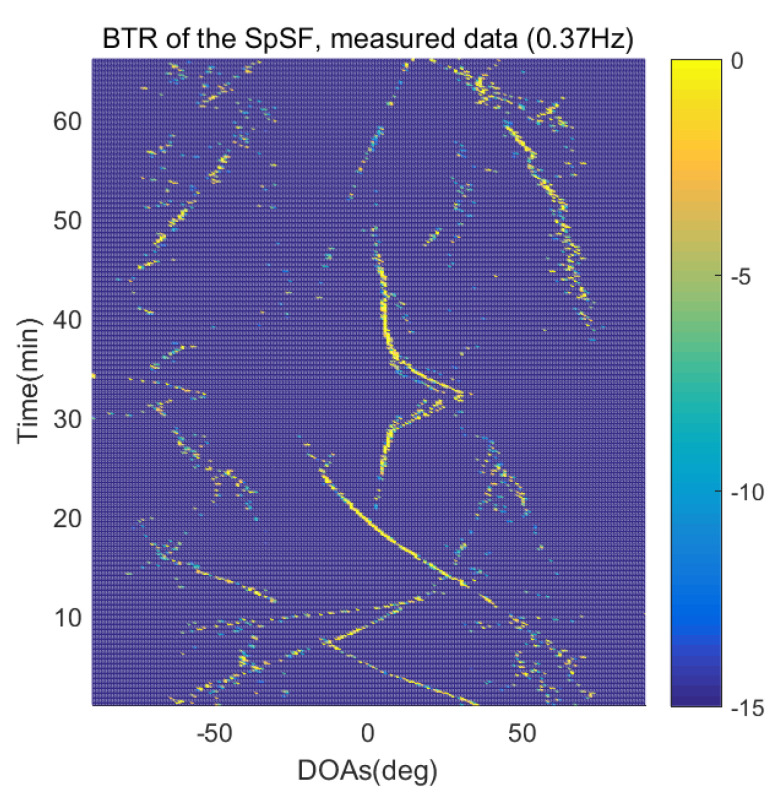
Bearing time records of the SpSF: 66 min, signal frequency = 0.37 Hz.

**Figure 21:**
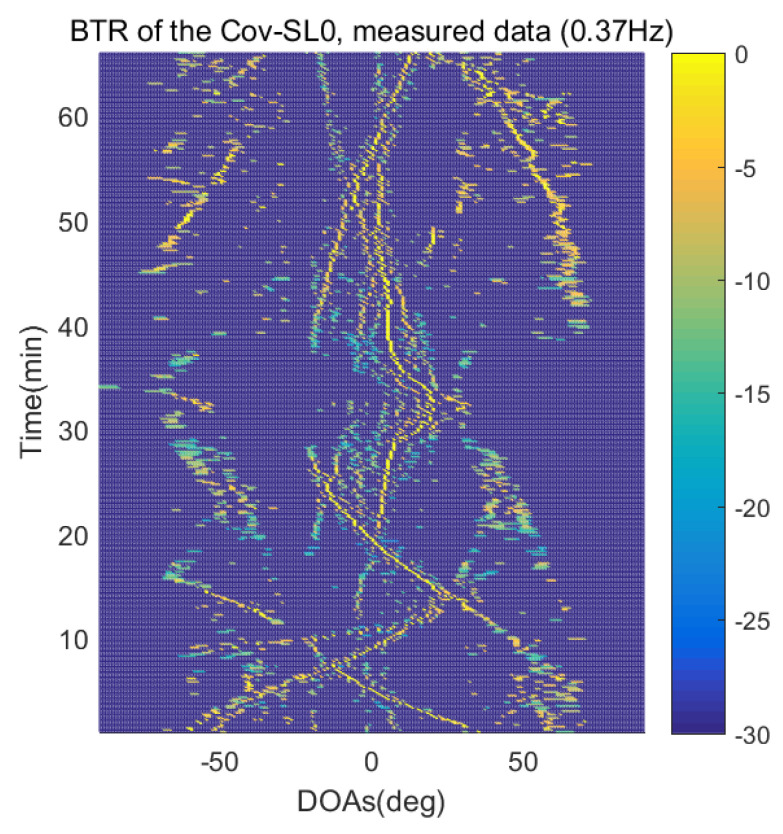
Bearing time records of the proposed scheme: 66 min, signal frequency = 0.37 Hz.

**Figure 22:**
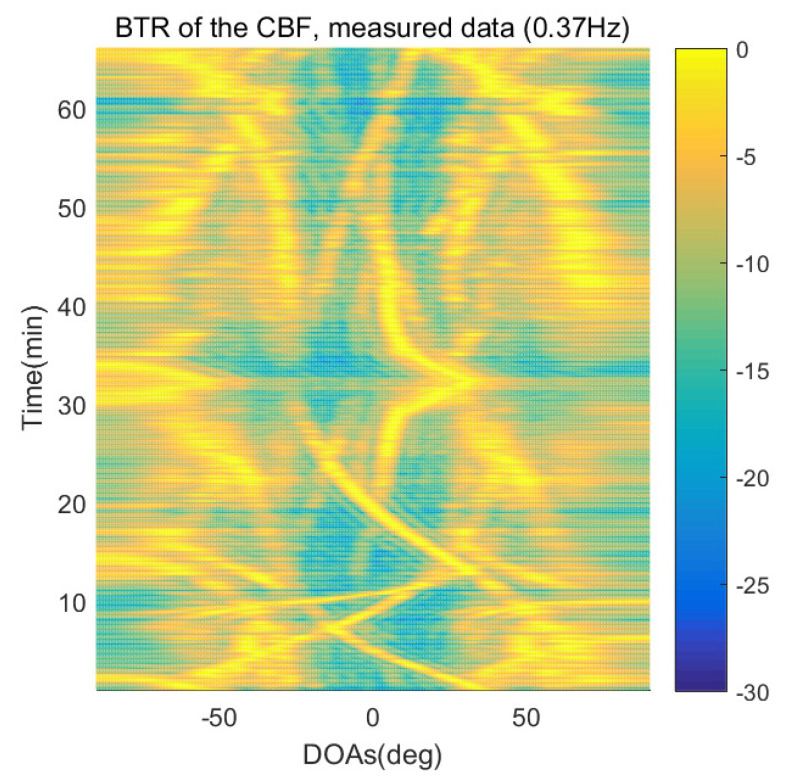
Bearing time records of the CBF: 66 min, signal frequency = 0.37 Hz.

**Figure 23:**
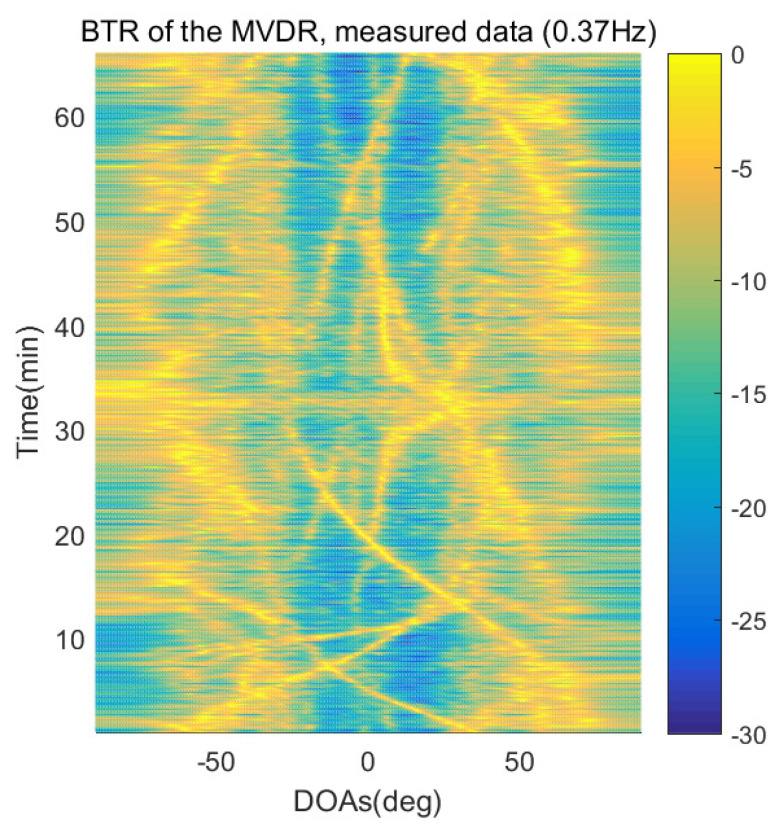
Bearing time records of the MVDR: 66 min, signal frequency = 0.37 Hz.

In Figure 20, Figure 21, Figure 22 and Figure 23, the performance of the proposed scheme, which is the SpSF, the conventional beamforming algorithm and the MVDR are shown when the signal frequency, which is 0.37 Hz, has a signal level that is 20 dB lower than the pilot signal frequency.

In Figure 16, Figure 17, Figure 18, Figure 19, Figure 20, Figure 21, Figure 22 and Figure 23, the compressive-sensing-based covariance-fitting methods can distinguish the trajectory of the moving signal source, and also the moving trajectories of other ships around it with high resolution. In the beamforming-based methods, the resolutions are significantly lower than the compressive-sensing methods, so the moving trajectories of the moving signal source and the other ships are unclear. These results verify that the DOA estimation performance of the proposed scheme is superior to that of the conventional beamforming algorithm and the MVDR. In Figure 16, Figure 17, Figure 20 and Figure 21, The proposed scheme and the SpSF algorithm have practically the same performance regarding the DOA estimation.

However, when comparing the BTRs of the SpSF algorithm and that of the beamforming methods, it can be seen that a lot of information about the moving trajectories of the other ships were removed from the BTRS of the SpSF algorithm. On the other hand, the BTRs of the proposed scheme preserve more moving trajectories of the other ships than that of the SpSF algorithm. It is verified through Figure 16, Figure 17, Figure 18, Figure 19, Figure 20, Figure 21, Figure 22 and Figure 23 that the proposed scheme is more appropriate than the SpSF algorithm when the DOAs of the target and the other signal sources must be simultaneously estimated.

## 6. Conclusions

In order to improve the performance of the conventional smoothed $l_{0}$ norm method that performs sparse recovery using a single sample, the compressive-sensing-based covariance-fitting smoothed $l_{0}$ norm method was proposed in this paper. The conventional method was extended based on the covariance-fitting method. By using the covariance matrix, it is possible to solve the problem with a large number of optimization variables when using multiple samples. The cost function and the null-projection term for the covariance fitting was presented.

The advantages of the proposed scheme in terms of DOA estimation performance and computational complexity were verified through the comparison simulations of the DOA estimation performance and the operation time between the conventional smoothed $l_{0}$ norm method, while multiple snapshots-based smoothed $l_{0}$ norm method, and the proposed scheme.

The DOA estimation performance of the proposed algorithm was shown by comparing the DOA estimation performance and resolution between the beamforming-based angle of arrival estimation algorithm, the convex relaxation-based SpSF algorithm, and the proposed algorithm using various simulation parameters. In addition, the computational efficiency of the proposed algorithm was shown through the operation time comparison simulation between the convex relaxation-based SpSF algorithm and the proposed algorithm. To verify the performance of the proposed algorithm, the results of the path estimation experiment of the moving signal source based on measured data were shown.

Furthermore, if only the dominant elements of the covariance matrix using the decomposition method, as in [19], are used, then the optimization performance of the proposed scheme can be further improved due to the reduction in computational complexity.

The following abbreviations are used in this manuscript:

## Figures and Tables

**Table 1:** Parameters of the measured data.

Parameter	Value
The number of sensors	15
Sampling frequency	2.625 Hz
Design frequencies	0.128 Hz, 0.256 Hz, 0.512 Hz, and 1.025 Hz
Search range of DOA	$-90^{{}^{\circ}}:1^{{}^{\circ}}:90^{{}^{\circ}}$
The pilot signal frequency	1 Hz
Signal frequencies	0.142 Hz, 0.37 Hz

## Data Availability

Not applicable.
